# Supplementary material for: Inference of stress-strength reliability for two-parameter of exponentiated Gumbel distribution based on lower record values
Source: PLoS One. 2021 Apr 2;16(4):e0249028. doi: 10.1371/journal.pone.0249028 (PMC8018638; doi:10.1371/journal.pone.0249028)
Supplement: S1 Dataset — (DOCX) [file pone.0249028.s001.docx]

In this paper, two datasets have been used as real strength data. As stated in section 4.3 Real Data Analysis, these datasets can be reached on page 574 of the book "Statistical Models and Methods for Lifetime Data" by Jerald F. Lawless in 2011. So, to reproduce our findings, one can refer to the above-mentioned book. Therefore, the findings of our manuscript can be replicated using the information provided in our manuscript. To ensure that everyone can access those data, it has been provided as below (these data are included in the paper as well):

The real datasets which were reported in Lawless (2011). Dataset 1 ($x$) and Dataset 2 ($y$) correspond to 35.0 and 35.5 stress level, respectively.

| Dataset 1 ($x$) | | | | |  | Dataset 2 ($y$) | | | | |
| --- | --- | --- | --- | --- | --- | --- | --- | --- | --- | --- |
| 230 | 169 | 178 | 271 | 129 |  | 156 | 173 | 125 | 852 | 559 |
| 568 | 115 | 280 | 305 | 326 |  | 442 | 168 | 286 | 261 | 227 |
| 1101 | 285 | 734 | 177 | 493 |  | 285 | 253 | 166 | 133 | 309 |
| 218 | 342 | 431 | 143 | 381 |  | 247 | 112 | 202 | 365 | 702 |

Figure 1 contains the proposal and posterior density functions of the scale parameter. The values are as below:

| **Posterior** | **Normal Density** |
| --- | --- |
| 3.84E-10 | 2.260985 |
| 2.36E-08 | 2.33221 |
| 2.59E-07 | 2.405302 |
| 1.40E-06 | 2.480298 |
| 5.15E-06 | 2.557233 |
| 1.48E-05 | 2.636141 |
| 3.60E-05 | 2.717061 |
| 7.72E-05 | 2.800026 |
| 0.000151 | 2.885074 |
| 0.000274 | 2.972241 |
| 0.000468 | 3.061563 |
| 0.00076 | 3.153077 |
| 0.001185 | 3.246819 |
| 0.001783 | 3.342825 |
| 0.002603 | 3.441132 |
| 0.0037 | 3.541778 |
| 0.005139 | 3.644797 |
| 0.006991 | 3.750227 |
| 0.009337 | 3.858103 |
| 0.012267 | 3.968463 |
| 0.015879 | 4.081341 |
| 0.020279 | 4.196774 |
| 0.025584 | 4.314798 |
| 0.031917 | 4.435449 |
| 0.039411 | 4.55876 |
| 0.048206 | 4.684767 |
| 0.058451 | 4.813506 |
| 0.070302 | 4.945009 |
| 0.083923 | 5.079311 |
| 0.099485 | 5.216446 |
| 0.117163 | 5.356446 |
| 0.137141 | 5.499344 |
| 0.159608 | 5.645172 |
| 0.184757 | 5.793961 |
| 0.212787 | 5.945744 |
| 0.2439 | 6.100549 |
| 0.278303 | 6.258407 |
| 0.316205 | 6.419346 |
| 0.357817 | 6.583395 |
| 0.403353 | 6.750582 |
| 0.453027 | 6.920933 |
| 0.507056 | 7.094475 |
| 0.565655 | 7.271231 |
| 0.629039 | 7.451227 |
| 0.697424 | 7.634486 |
| 0.771021 | 7.82103 |
| 0.850043 | 8.01088 |
| 0.934696 | 8.204057 |
| 1.025186 | 8.400579 |
| 1.121714 | 8.600465 |
| 1.224477 | 8.803731 |
| 1.333667 | 9.010394 |
| 1.44947 | 9.220466 |
| 1.572068 | 9.433963 |
| 1.701634 | 9.650895 |
| 1.838337 | 9.871272 |
| 1.982337 | 10.0951 |
| 2.133786 | 10.3224 |
| 2.292829 | 10.55316 |
| 2.459603 | 10.7874 |
| 2.634235 | 11.02511 |
| 2.816842 | 11.2663 |
| 3.007534 | 11.51097 |
| 3.206408 | 11.75912 |
| 3.413553 | 12.01073 |
| 3.629046 | 12.26582 |
| 3.852956 | 12.52436 |
| 4.085338 | 12.78636 |
| 4.326237 | 13.05179 |
| 4.575686 | 13.32066 |
| 4.833709 | 13.59294 |
| 5.100316 | 13.86862 |
| 5.375505 | 14.14768 |
| 5.659263 | 14.4301 |
| 5.951566 | 14.71586 |
| 6.252377 | 15.00493 |
| 6.561646 | 15.29729 |
| 6.879313 | 15.59291 |
| 7.205305 | 15.89176 |
| 7.539538 | 16.1938 |
| 7.881916 | 16.49901 |
| 8.232329 | 16.80735 |
| 8.590658 | 17.11877 |
| 8.956772 | 17.43324 |
| 9.330529 | 17.75071 |
| 9.711775 | 18.07114 |
| 10.10034 | 18.39448 |
| 10.49606 | 18.72068 |
| 10.89875 | 19.04969 |
| 11.3082 | 19.38145 |
| 11.72421 | 19.7159 |
| 12.14656 | 20.053 |
| 12.57504 | 20.39267 |
| 13.0094 | 20.73486 |
| 13.44941 | 21.07949 |
| 13.89481 | 21.42651 |
| 14.34535 | 21.77583 |
| 14.80075 | 22.12739 |
| 15.26075 | 22.48111 |
| 15.72505 | 22.83693 |
| 16.19339 | 23.19474 |
| 16.66545 | 23.55448 |
| 17.14095 | 23.91607 |
| 17.61958 | 24.27941 |
| 18.10102 | 24.64442 |
| 18.58498 | 25.01101 |
| 19.07112 | 25.37909 |
| 19.55913 | 25.74856 |
| 20.04868 | 26.11933 |
| 20.53946 | 26.4913 |
| 21.03112 | 26.86436 |
| 21.52335 | 27.23843 |
| 22.0158 | 27.61339 |
| 22.50815 | 27.98913 |
| 23.00006 | 28.36556 |
| 23.49121 | 28.74255 |
| 23.98125 | 29.12001 |
| 24.46986 | 29.49782 |
| 24.95671 | 29.87585 |
| 25.44147 | 30.25401 |
| 25.92381 | 30.63217 |
| 26.40342 | 31.0102 |
| 26.87996 | 31.388 |
| 27.35313 | 31.76543 |
| 27.8226 | 32.14239 |
| 28.28808 | 32.51873 |
| 28.74925 | 32.89434 |
| 29.20582 | 33.26909 |
| 29.65748 | 33.64285 |
| 30.10395 | 34.0155 |
| 30.54495 | 34.3869 |
| 30.98019 | 34.75692 |
| 31.40941 | 35.12544 |
| 31.83233 | 35.49232 |
| 32.2487 | 35.85742 |
| 32.65828 | 36.22063 |
| 33.0608 | 36.58179 |
| 33.45604 | 36.94079 |
| 33.84376 | 37.29747 |
| 34.22375 | 37.65172 |
| 34.59578 | 38.0034 |
| 34.95966 | 38.35237 |
| 35.31519 | 38.69849 |
| 35.66217 | 39.04164 |
| 36.00043 | 39.38168 |
| 36.32979 | 39.71847 |
| 36.65009 | 40.05188 |
| 36.96118 | 40.38178 |
| 37.26291 | 40.70804 |
| 37.55514 | 41.03052 |
| 37.83774 | 41.3491 |
| 38.1106 | 41.66364 |
| 38.37361 | 41.97401 |
| 38.62666 | 42.28009 |
| 38.86967 | 42.58174 |
| 39.10254 | 42.87885 |
| 39.3252 | 43.17129 |
| 39.53759 | 43.45893 |
| 39.73966 | 43.74164 |
| 39.93134 | 44.01933 |
| 40.11261 | 44.29185 |
| 40.28343 | 44.55909 |
| 40.44378 | 44.82095 |
| 40.59364 | 45.0773 |
| 40.73301 | 45.32803 |
| 40.86188 | 45.57304 |
| 40.98028 | 45.81221 |
| 41.0882 | 46.04544 |
| 41.18569 | 46.27263 |
| 41.27277 | 46.49367 |
| 41.34948 | 46.70847 |
| 41.41587 | 46.91694 |
| 41.472 | 47.11897 |
| 41.51791 | 47.31447 |
| 41.55368 | 47.50337 |
| 41.57939 | 47.68557 |
| 41.59511 | 47.86098 |
| 41.60093 | 48.02954 |
| 41.59694 | 48.19116 |
| 41.58324 | 48.34577 |
| 41.55993 | 48.4933 |
| 41.52713 | 48.63368 |
| 41.48493 | 48.76684 |
| 41.43346 | 48.89273 |
| 41.37285 | 49.01129 |
| 41.30322 | 49.12245 |
| 41.2247 | 49.22618 |
| 41.13743 | 49.32242 |
| 41.04155 | 49.41113 |
| 40.9372 | 49.49226 |
| 40.82452 | 49.56578 |
| 40.70367 | 49.63165 |
| 40.5748 | 49.68985 |
| 40.43807 | 49.74034 |
| 40.29362 | 49.7831 |
| 40.14164 | 49.81812 |
| 39.98226 | 49.84537 |
| 39.81567 | 49.86485 |
| 39.64203 | 49.87653 |
| 39.4615 | 49.88043 |
| 39.27426 | 49.87653 |
| 39.08047 | 49.86485 |
| 38.88032 | 49.84537 |
| 38.67396 | 49.81812 |
| 38.46159 | 49.7831 |
| 38.24336 | 49.74034 |
| 38.01946 | 49.68985 |
| 37.79006 | 49.63165 |
| 37.55534 | 49.56578 |
| 37.31548 | 49.49226 |
| 37.07064 | 49.41113 |
| 36.821 | 49.32242 |
| 36.56674 | 49.22618 |
| 36.30803 | 49.12245 |
| 36.04505 | 49.01129 |
| 35.77796 | 48.89273 |
| 35.50694 | 48.76684 |
| 35.23217 | 48.63368 |
| 34.9538 | 48.4933 |
| 34.67201 | 48.34577 |
| 34.38696 | 48.19116 |
| 34.09882 | 48.02954 |
| 33.80775 | 47.86098 |
| 33.51391 | 47.68557 |
| 33.21747 | 47.50337 |
| 32.91859 | 47.31447 |
| 32.61741 | 47.11897 |
| 32.31409 | 46.91694 |
| 32.00879 | 46.70847 |
| 31.70166 | 46.49367 |
| 31.39283 | 46.27263 |
| 31.08247 | 46.04544 |
| 30.77071 | 45.81221 |
| 30.4577 | 45.57304 |
| 30.14356 | 45.32803 |
| 29.82845 | 45.0773 |
| 29.51249 | 44.82095 |
| 29.19581 | 44.55909 |
| 28.87854 | 44.29185 |
| 28.56081 | 44.01933 |
| 28.24275 | 43.74164 |
| 27.92446 | 43.45893 |
| 27.60608 | 43.17129 |
| 27.28771 | 42.87885 |
| 26.96948 | 42.58174 |
| 26.65148 | 42.28009 |
| 26.33383 | 41.97401 |
| 26.01663 | 41.66364 |
| 25.69999 | 41.3491 |
| 25.38399 | 41.03052 |
| 25.06875 | 40.70804 |
| 24.75436 | 40.38178 |
| 24.4409 | 40.05188 |
| 24.12846 | 39.71847 |
| 23.81714 | 39.38168 |
| 23.507 | 39.04164 |
| 23.19815 | 38.69849 |
| 22.89064 | 38.35237 |
| 22.58456 | 38.0034 |
| 22.27999 | 37.65172 |
| 21.97699 | 37.29747 |
| 21.67562 | 36.94079 |
| 21.37596 | 36.58179 |
| 21.07807 | 36.22063 |
| 20.78201 | 35.85742 |
| 20.48783 | 35.49232 |
| 20.1956 | 35.12544 |
| 19.90536 | 34.75692 |
| 19.61716 | 34.3869 |
| 19.33106 | 34.0155 |
| 19.04711 | 33.64285 |
| 18.76534 | 33.26909 |
| 18.48579 | 32.89434 |
| 18.20852 | 32.51873 |
| 17.93354 | 32.14239 |
| 17.66091 | 31.76543 |
| 17.39066 | 31.388 |
| 17.12281 | 31.0102 |
| 16.8574 | 30.63217 |
| 16.59445 | 30.25401 |
| 16.33399 | 29.87585 |
| 16.07604 | 29.49782 |
| 15.82063 | 29.12001 |
| 15.56777 | 28.74255 |
| 15.3175 | 28.36556 |
| 15.06981 | 27.98913 |
| 14.82473 | 27.61339 |
| 14.58227 | 27.23843 |
| 14.34244 | 26.86436 |
| 14.10526 | 26.4913 |
| 13.87073 | 26.11933 |
| 13.63887 | 25.74856 |
| 13.40967 | 25.37909 |
| 13.18314 | 25.01101 |
| 12.95929 | 24.64442 |
| 12.73811 | 24.27941 |
| 12.51962 | 23.91607 |
| 12.30381 | 23.55448 |
| 12.09067 | 23.19474 |
| 11.88021 | 22.83693 |
| 11.67242 | 22.48111 |
| 11.4673 | 22.12739 |
| 11.26484 | 21.77583 |
| 11.06504 | 21.42651 |
| 10.86788 | 21.07949 |
| 10.67337 | 20.73486 |
| 10.48148 | 20.39267 |
| 10.29221 | 20.053 |
| 10.10555 | 19.7159 |
| 9.921485 | 19.38145 |
| 9.740005 | 19.04969 |
| 9.561094 | 18.72068 |
| 9.38474 | 18.39448 |
| 9.210926 | 18.07114 |
| 9.039638 | 17.75071 |
| 8.870859 | 17.43324 |
| 8.704574 | 17.11877 |
| 8.540763 | 16.80735 |
| 8.379411 | 16.49901 |
| 8.220498 | 16.1938 |
| 8.064006 | 15.89176 |
| 7.909916 | 15.59291 |
| 7.758208 | 15.29729 |
| 7.608861 | 15.00493 |
| 7.461856 | 14.71586 |
| 7.317172 | 14.4301 |
| 7.174787 | 14.14768 |
| 7.034681 | 13.86862 |
| 6.896831 | 13.59294 |
| 6.761215 | 13.32066 |
| 6.627812 | 13.05179 |
| 6.496598 | 12.78636 |
| 6.367552 | 12.52436 |
| 6.240649 | 12.26582 |
| 6.115868 | 12.01073 |
| 5.993185 | 11.75912 |
| 5.872576 | 11.51097 |
| 5.754018 | 11.2663 |
| 5.637488 | 11.02511 |
| 5.522962 | 10.7874 |
| 5.410416 | 10.55316 |
| 5.299826 | 10.3224 |
| 5.191169 | 10.0951 |
| 5.08442 | 9.871272 |
| 4.979556 | 9.650895 |
| 4.876553 | 9.433963 |
| 4.775387 | 9.220466 |
| 4.676033 | 9.010394 |
| 4.578469 | 8.803731 |
| 4.48267 | 8.600465 |
| 4.388613 | 8.400579 |
| 4.296274 | 8.204057 |
| 4.205629 | 8.01088 |
| 4.116655 | 7.82103 |
| 4.029327 | 7.634486 |
| 3.943624 | 7.451227 |
| 3.859522 | 7.271231 |
| 3.776997 | 7.094475 |
| 3.696026 | 6.920933 |
| 3.616587 | 6.750582 |
| 3.538657 | 6.583395 |
| 3.462213 | 6.419346 |
| 3.387232 | 6.258407 |
| 3.313693 | 6.100549 |
| 3.241574 | 5.945744 |
| 3.170852 | 5.793961 |
| 3.101505 | 5.645172 |
| 3.033512 | 5.499344 |
| 2.966852 | 5.356446 |
| 2.901502 | 5.216446 |
| 2.837443 | 5.079311 |
| 2.774653 | 4.945009 |
| 2.713111 | 4.813506 |
| 2.652797 | 4.684767 |
| 2.593691 | 4.55876 |
| 2.535772 | 4.435449 |
| 2.479021 | 4.314798 |
| 2.423418 | 4.196774 |
| 2.368944 | 4.081341 |
| 2.315579 | 3.968463 |
| 2.263304 | 3.858103 |
| 2.2121 | 3.750227 |
| 2.161949 | 3.644797 |
| 2.112833 | 3.541778 |
| 2.064733 | 3.441132 |
| 2.017631 | 3.342825 |
| 1.971509 | 3.246819 |
| 1.926351 | 3.153077 |
| 1.882138 | 3.061563 |
| 1.838854 | 2.972241 |
| 1.796481 | 2.885074 |
| 1.755004 | 2.800026 |
| 1.714406 | 2.717061 |
| 1.67467 | 2.636141 |
| 1.635781 | 2.557233 |
| 1.597723 | 2.480298 |
| 1.56048 | 2.405302 |
| 1.524037 | 2.33221 |
| 1.488379 | 2.260985 |
| 1.453492 | 2.191593 |
| 1.41936 | 2.123999 |
| 1.385969 | 2.058168 |
| 1.353305 | 1.994066 |
| 1.321353 | 1.931658 |
| 1.290101 | 1.870911 |
| 1.259535 | 1.811792 |
| 1.22964 | 1.754266 |
| 1.200405 | 1.698302 |
| 1.171815 | 1.643866 |
| 1.143859 | 1.590926 |
| 1.116524 | 1.539451 |
| 1.089797 | 1.489408 |
| 1.063666 | 1.440767 |
| 1.038119 | 1.393497 |
| 1.013146 | 1.347567 |
| 0.988733 | 1.302947 |
| 0.96487 | 1.259608 |
| 0.941545 | 1.21752 |
| 0.918748 | 1.176655 |
| 0.896467 | 1.136984 |
| 0.874693 | 1.098478 |
| 0.853414 | 1.061111 |
| 0.832621 | 1.024854 |
| 0.812303 | 0.989682 |
| 0.792451 | 0.955568 |
| 0.773054 | 0.922485 |
| 0.754104 | 0.890409 |
| 0.73559 | 0.859314 |
| 0.717504 | 0.829175 |
| 0.699837 | 0.799968 |
| 0.68258 | 0.771669 |
| 0.665723 | 0.744255 |
| 0.649259 | 0.717703 |
| 0.63318 | 0.69199 |
| 0.617476 | 0.667094 |
| 0.60214 | 0.642993 |
| 0.587163 | 0.619666 |
| 0.572539 | 0.597092 |
| 0.55826 | 0.575251 |
| 0.544317 | 0.554122 |
| 0.530704 | 0.533685 |
| 0.517413 | 0.513922 |
| 0.504437 | 0.494813 |
| 0.491771 | 0.476341 |
| 0.479405 | 0.458487 |
| 0.467335 | 0.441232 |
| 0.455553 | 0.424561 |
| 0.444053 | 0.408456 |
| 0.432829 | 0.3929 |
| 0.421874 | 0.377878 |
| 0.411183 | 0.363373 |
| 0.40075 | 0.349371 |
| 0.390568 | 0.335855 |
| 0.380633 | 0.322812 |
| 0.370938 | 0.310228 |
| 0.361479 | 0.298087 |
| 0.352249 | 0.286376 |
| 0.343244 | 0.275083 |
| 0.334459 | 0.264193 |
| 0.325888 | 0.253695 |
| 0.317527 | 0.243576 |
| 0.30937 | 0.233825 |
| 0.301414 | 0.224428 |
| 0.293653 | 0.215376 |
| 0.286083 | 0.206656 |
| 0.278699 | 0.198258 |
| 0.271498 | 0.190172 |
| 0.264475 | 0.182388 |
| 0.257625 | 0.174894 |
| 0.250946 | 0.167682 |
| 0.244432 | 0.160743 |
| 0.23808 | 0.154066 |
| 0.231887 | 0.147644 |
| 0.225848 | 0.141468 |
| 0.219959 | 0.135528 |
| 0.214218 | 0.129818 |
| 0.208621 | 0.124329 |
| 0.203164 | 0.119053 |
| 0.197844 | 0.113984 |
| 0.192657 | 0.109113 |
| 0.187602 | 0.104434 |
| 0.182673 | 0.09994 |
| 0.17787 | 0.095625 |
| 0.173187 | 0.091481 |
| 0.168623 | 0.087504 |
| 0.164175 | 0.083686 |
| 0.15984 | 0.080022 |
| 0.155615 | 0.076507 |
| 0.151497 | 0.073135 |
| 0.147485 | 0.0699 |
| 0.143574 | 0.066799 |
| 0.139764 | 0.063824 |
| 0.136051 | 0.060973 |
| 0.132433 | 0.05824 |
| 0.128908 | 0.055621 |
| 0.125473 | 0.053111 |
| 0.122127 | 0.050707 |
| 0.118866 | 0.048403 |
| 0.11569 | 0.046198 |
| 0.112596 | 0.044086 |

Figure 2 contains the empirical CDF points. The values are as below:

| **Dataset 1 (x)** | **Empirical CDF** |
| --- | --- |
| 115 | 0.0719212279684049 |
| 129 | 0.0923341012962292 |
| 143 | 0.115762543390081 |
| 169 | 0.166683622351636 |
| 177 | 0.184075607500274 |
| 178 | 0.186300270395955 |
| 218 | 0.282591210958021 |
| 230 | 0.313419347009354 |
| 271 | 0.420482095983728 |
| 280 | 0.443723918424522 |
| 285 | 0.456520943674426 |
| 305 | 0.506617387685895 |
| 326 | 0.556817405913310 |
| 342 | 0.593068719072731 |
| 381 | 0.673196824095878 |
| 431 | 0.757948464670944 |
| 493 | 0.836781793160388 |
| 568 | 0.900830620381315 |
| 734 | 0.968495854677683 |
| 1101 | 0.997659038479823 |

Figure 3 contains the empirical CDF points. The values are as below:

| **Dataset 2 (y)** | **Empirical CDF** |
| --- | --- |
| 112 | 0.0789969342415915 |
| 125 | 0.102939982805443 |
| 133 | 0.119479770907944 |
| 156 | 0.174047431730863 |
| 166 | 0.200610198121388 |
| 168 | 0.206098688035014 |
| 173 | 0.220052491651908 |
| 202 | 0.306024196738187 |
| 227 | 0.383634788334955 |
| 247 | 0.445424477559749 |
| 253 | 0.463641921846360 |
| 261 | 0.487595187256323 |
| 285 | 0.556489633395641 |
| 286 | 0.559248229225808 |
| 309 | 0.619859790763133 |
| 365 | 0.742611428530314 |
| 442 | 0.856432055632269 |
| 559 | 0.944110243253334 |
| 702 | 0.983022936371675 |
| 852 | 0.995207195497212 |
