# Supplementary material for: Inference of stress-strength reliability for two-parameter of exponentiated Gumbel distribution based on lower record values
Source: PLoS One. 2021 Apr 2;16(4):e0249028. doi: 10.1371/journal.pone.0249028 (PMC8018638; doi:10.1371/journal.pone.0249028)
Supplement: Attachment — Submitted filename: Response to Reviewers.docx [file pone.0249028.s002.docx]

**RESPONSES TO THE REVIEWERS’ COMMENTS**

We would like to express our gratitude to the Editor and Reviewers for their valuable comments and contributions to improve our study. We have replied to the comments point-by-point and revised the manuscript by considering the suggestions and recommendations.

Here are the answers to reviewers’ suggestions and **revised parts are shown as track changes** in the manuscript.

***Reviewers' comments:***

***Comments to the Author***

***Comment 1:*** *Is the manuscript technically sound, and do the data support the conclusions?*

*The manuscript must describe a technically sound piece of scientific research with data that supports the conclusions. Experiments must have been conducted rigorously, with appropriate controls, replication, and sample sizes. The conclusions must be drawn appropriately based on the data presented.*

***Reviewer #1:*** *Yes*

***Reviewer #2:*** *Partly*

***Comment 2:*** *Has the statistical analysis been performed appropriately and rigorously?*

***Reviewer #1:*** *Yes*

***Reviewer #2:*** *Yes*

***Comment 3:*** *Have the authors made all data underlying the findings in their manuscript fully available?*

*The* [*PLOS Data policy*](http://www.plosone.org/static/policies.action#sharing) *requires authors to make all data underlying the findings described in their manuscript fully available without restriction, with rare exception (please refer to the Data Availability Statement in the manuscript PDF file). The data should be provided as part of the manuscript or its supporting information, or deposited to a public repository. For example, in addition to summary statistics, the data points behind means, medians and variance measures should be available. If there are restrictions on publicly sharing data—e.g. participant privacy or use of data from a third party—those must be specified.*

***Reviewer #1:*** *Yes*

***Reviewer #2:*** *Yes*

***Comment 4:*** *Is the manuscript presented in an intelligible fashion and written in standard English?*

*PLOS ONE does not copyedit accepted manuscripts, so the language in submitted articles must be clear, correct, and unambiguous. Any typographical or grammatical errors should be corrected at revision, so please note any specific errors here.*

***Reviewer #1:*** *Yes*

***Reviewer #2:*** *Yes*

***Comment 5:*** *Review Comments to the Author*

*Please use the space provided to explain your answers to the questions above. You may also include additional comments for the author, including concerns about dual publication, research ethics, or publication ethics. (Please upload your review as an attachment if it exceeds 20,000 characters)*

***Reviewer #1:*** *This paper proposes a Two-Parameter of exponentiated Gumbel distribution for modeling stress-strength reliability, based on lower record values. The research topic is interesting and worth of investigation. The proposed model is promising and its strengths have been demonstrated by two real-word datasets. A minor suggestion is that more works on Bayesian estimation and its engineer applications should be referred, such as:*

*A Bayesian spatial random parameters Tobit model for analyzing crash rates on roadway segments. Accident Analysis and Prevention, 2017, 100: 37-43.*

*Bayesian spatial-temporal model for the main and interaction effects of roadway and weather characteristics on freeway crash incidence. Accident Analysis and Prevention, 2019, 132, 105249.*

*Spatial joint analysis for zonal daytime and nighttime crash frequencies using a Bayesian bivariate conditional autoregressive model. Journal of Transportation Safety and Security, 2020, 12(4): 566-585.*

*The authors are also suggested to add a Conclusion section to draw some remarkable findings and present some directions for future research.*

**Answer:** In line with the referee’s comment, reference list is revised. Also, Conclusion section has been added.

***Reviewer #2:*** *The topic of this paper is interesting. The methods sound. The results are meaningful and useful. There are some suggestions to improve this paper.*

*1. More reference of maximum likelihood is needed. For example, the following ones.*

*[1] “Investigating the Differences of Single- and Multi-vehicle Accident Probability Using Mixed Logit Model", Journal of Advanced Transportation, 2018, UNSP 2702360.*

*[2] “Injury severities of truck drivers in single- and multi-vehicle accidents on rural highway”, Accident Analysis and Prevention, 2011, 43(5), 1677-1688.*

*[3] Analysis of hourly crash likelihood using unbalanced panel data mixed logit model and real-time driving environmental big data. 2018, JOURNAL OF SAFETY RESEARCH. 65: 153-159.*

*[2] A conclusion part is needed to summarize this paper.*

**Answer:** In line with the referee’s comment, reference list is revised. Also, Conclusion section has been added.

***Comment* 6:** *PLOS authors have the option to publish the peer review history of their article (*[*what does this mean?*](https://journals.plos.org/plosone/s/editorial-and-peer-review-process#loc-peer-review-history)*). If published, this will include your full peer review and any attached files.*

*If you choose “no”, your identity will remain anonymous but your review may still be made public.*

***Do you want your identity to be public for this peer review?*** *For information about this choice, including consent withdrawal, please see our* [*Privacy Policy*](https://www.plos.org/privacy-policy)*.*

***Reviewer #1:*** *No*

***Reviewer #2:*** *No*

Dear Editor,

Firstly, we want to thank you for your time and considerations. We have shown the revised parts as track changes in the file that is named as “Revised Manuscript with Track Changes”. We have modified the manuscript based on all required changes you mentioned, including:

1- We have referred to Bayesian works in our work, especially those references mentioned by reviewers.
2- We have referred to more references of maximum likelihood works in our work, especially those you mentioned.
3- We have added a Conclusion section to draw some remarkable findings and present some directions for future research.

We would like to present the updated version of our manuscript for evaluation. We are looking forward to getting your final decision on our work.

Best regards,
